# Matrix Remodeling-Associated Protein 8 as a Novel Indicator Contributing to Glioma Immune Response by Regulating Ferroptosis

**DOI:** 10.3389/fimmu.2022.834595

**Published:** 2022-02-24

**Authors:** Zhijie Xu, Xi Chen, Liying Song, Fang Yuan, Yuanliang Yan

**Affiliations:** ^1^Department of Pathology, Xiangya Hospital, Central South University, Changsha, China; ^2^Department of Pharmacy, Xiangya Hospital, Central South University, Changsha, China; ^3^National Clinical Research Center for Geriatric Disorders, Xiangya Hospital, Central South University, Changsha, China; ^4^Department of Pharmacy, The Third Xiangya Hospital, Central South University, Changsha, China; ^5^Department of Plastic Surgery, The Third Xiangya Hospital, Central South University, Changsha, China

**Keywords:** MXRA8, immune response, ferroptosis, glioma, prognosis

## Abstract

Glioma is a highly malignant brain tumor with a poor survival rate. Novel biomarkers that act as prompt indicators of glioma are urgently needed. In this study, we identified and validated prognosis-related differentially expressed genes by datasets of glioma in the GEO and TCGA databases. Ferroptosis is a newly recognized process of cell death playing a vital role in cancer biology. Pearson correlation coefficient were used to discovery the prognosis-related genes which have the highest correlation with ferroptosis. Matrix remodeling-associated protein 8 (MXRA8) was identified as a novel prognosis indicator which may be involved in ferroptosis. The expression of MXRA8 was significantly higher in glioma compared with normal brain tissue, and increased expression of MXRA8 was associated with unfavorable survivals. Furthermore, *in vitro* analysis showed that knockdown of MXRA8 inhibited the cell viability in T98G and U251 cells and increased the sensitivity of glioma cells to temozolomide. We further observed that downregulation of MXRA8 elevated the levels of intracellular ferrous iron and lipid peroxidation, accompanied by upregulation of NCOA4 and suppression of FTH1. Moreover, co-expression analyses showed that GO term and KEGG pathways were mainly enriched in immunity-related pathways, such as neutrophil-related immunity, adaptive immune response, and cytokine binding. Through ssGSEA algorithm and TISIDB database, immunological analyses showed that MXRA8 was significantly correlated with various immune infiltration cells including NK cells, macrophages, and neutrophils. Meanwhile, MXRA8 was also associated with chemokines and multiple immunoinhibitory molecules, such as TGF-β1, IL-10, PD-L1, and CTLA4. We also found that MXRA8 was positively associated with immune infiltration score, and patients with higher immune score underwent worse overall survivals. Moreover, IHC staining indicated a highly positive correlation of MXRA8 with a macrophage marker CSF1R. The co-cultured models of glioma cells and M2 macrophages showed MXRA8 knockdown glioma cells alleviated the infiltration of M2 macrophage, while the reduced M2 macrophage infiltration generated by MXRA8 could be rescued by Fer-1 treatment. These results suggest that MXRA8 promotes glioma progression and highlight the pivotal role of MXRA8 in ferroptosis and immune microenvironment of glioma. Therefore, MXRA8 may serve as a novel prognostic marker and therapeutic target for glioma.

## Introduction

Glioma is one of the most common primary brain tumor, with an annual incidence of 3–8 per 100,000 population (1). As the most malignant subtype of glioma, glioblastoma multiforme (GBM) have an around 14 months of median survival (2, 3). Despite the availability of conventional treatments, such as surgical resection, adjuvant radiotherapy, and chemotherapy, the effects of prolonged survivals in GBM patients were still limited (4). Hence, exploring novel prognostic biomarkers for glioma therapy is crucial.

Ferroptosis, a newly discovered form of regulated cell death, involves an iron-dependent accumulation of lethal lipid peroxidation (5). Dysregulation of iron metabolism were thought to be a hallmark for ferroptosis and triggers intracellular reactive oxygen species (ROS) production by the Fenton reaction (6). Furthermore, disruption of glutathione metabolism system could also cause overload of lipid peroxidation, leading to extremely harmful cell damages in the process of ferroptosis (7). Over the past years, the essential role of ferroptosis in regulation of diverse physiological conditions and pathological progression has been identified in humans, and it has been gradually recognized as an adaptive process to render cancer cells vulnerable to death (8). Some studies have shown that tumor suppressor gene, TP53, could repress the synthesis of glutathione leading to ferroptosis promotion (9). In glioma, it has been reported that p53-dependent inhibition of SLC7A11, a unit of the glutamate-cystine antiporter required for ferroptosis induction, could deplete glutathione and elevate the levels of intracellular ferrous iron (Fe^2+^), H_2_O_2_, and lipid peroxidation (10). Furthermore, ferroptosis in glioma could be triggered by a battery of inducers, such as RSL3 and erastin, accompanied by suppression of proliferation and angiogenesis (11). Hence, induction of ferroptosis is emerging to be an effective strategy to eliminate glioma development, and the role of ferroptosis in glioma still needs to be further elucidated.

Emerging studies have established that immunotherapy could become an effective method by manipulating the immune system to recognize and attack cancer cells (12). Recently, the crosstalk between ferroptosis and antitumor immunity has been increasingly recognized. Study showed that ferroptosis could be integrated with immunotherapy through activated CD8+ T cells, by enhancing lipid peroxidation in cancers (13). Additionally, tumor cells treated by ferroptosis inhibit increased the resistance of a PD-L1 inhibitor (13). CD8+ T cell-derived IFN-γ cooperated with radiotherapy-activated ataxia-telangiectasia mutation (ATM) could induce ferroptosis in human fibrosarcoma cells and melanoma cells (14). These studies provoked a new insight of the relationship between ferroptosis activation and immune system regulation.

In the present study, we thus explored the differential expression of ferroptosis-related genes in glioma samples through the GEO datasets and TCGA database. We identified that Matrix remodeling-associated protein 8 (MXRA8) is highly expressed in glioma sample and is associated with poor prognosis of glioma. MXRA8 is reported to confer debilitating acute and chronic musculoskeletal disease, since it serves as an entry mediator for multiple arthritogenic alphaviruses (15). In terms of tumor progression, several studies have reported that high expressions of MXRA8 had poor overall survival (OS) in kidney renal clear cell carcinoma patients and is correlated with tumor microenvironment in esophageal squamous cell carcinoma (16). However, the underlying functions of MXRA8 in gliomas have not been elucidated. To further understand the role of MXRA8 in glioma, *in vitro* analysis is conducted and shows that knockdown of MXRA8 inhibited cell proliferations, increased the TMZ sensitivity, and induced ferroptosis in glioma. Further bioinformatics analysis revealed that MXRA8 function could be enriched in immune responses, and MXRA8 expression was positively associated with immunoinhibitory chemokines and checkpoints. Our data suggest that MXRA8 might act as a potential prognostic marker for glioma involving ferroptosis and immunity mediation.

## Materials and Methods

### The DEGs in Glioma Screened From the GEO and TCGA Datasets

The GEO (http://www.ncbi.nlm.nih.gov/geo) is an open-source platform for the storage of gene expression, chips, and microarray data. Two expression profiling datasets (GSE147352 and GSE59612) were, respectively, downloaded from the GEO database. The GSE147352 dataset includes 103 glioma and 15 normal brain tissues. The GSE59612 dataset includes 39 glioma and 17 nonneoplastic brain tissues. According to the setting cutoff criteria (fold change ≥2, *p* < 0.001), the differentially expressed genes (DEGs) between glioma and normal brain were identified. LASSO Cox regression was applied to predict the prognosis-related genes in glioma. For LASSO analysis, RNA-seq data and the latest clinical follow-up information were downloaded from the TCGA using GDC API (https://portal.gdc.cancer.gov/repository), including 606 RNA-seq data samples. The gene expression profiles were normalized using the scale method provided in the R package “limma”. To analyze the gene signature that contains the most helpful biomarkers for prognosis, R package “glmnet” was used and then the risk score of each sample in all the datasets was calculated through the signature (17). For survival analysis, samples were divided into high- and low-risk groups relying on their own median risk score by using R package “survival.” To verify the prognostic value of the MXRA8-based classifier, time-dependent (3-year, 5-year, and 10-year) receiver operating characteristics (ROC) were analyzed using the R package “timeROC” in the TCGA datasets (18).

### Clinical Feature Analysis

GEPIA2.0 (http://gepia.cancer-pku.cn/) (19) and UALCAN (http://ualcan.path.uab.edu/analysis.html) (20) were used to access mRNA data of glioma and normal specimens from the TCGA database. Furthermore, the clinical and survival analyses were obtained from the CGGA database (http://www.cgga.org.cn). In total, 325 samples and 693 samples of RNA sequencing data from the CGGA database 2 (http://www.cgga.org) were used for subtype analysis of the grade, IDH type, methylation status, age, and survivals. Clinical specimens were collected from glioma patients admitted for operation in Beijing Tiantan Hospital (21). Xiantao tool (https://www.xiantao.love/products) is a comprehensive bioinformatics analysis portal which can perform differential expression, enrichment analysis, interaction network, and clinical significance across pan-cancer types (22). Univariate regression analysis was performed to analyze the demographic characteristics of MXRA8 expression in glioma using the Xiantao tool.

### The GO and KEGG Pathway Analyses

LinkedOmics is an open-access portal that provides multiomics data across various cancer types with three analytical modules: LinkFinder, LinkInterpreter, and LinkCompare (23). The heatmaps of positively or negatively correlated genes with MXRA8 were analyzed with the LinkFinder module. Correlation analyses were performed using Spearman’s correlation test. The LinkInterpreter module was used to analyze the Gene Ontology (GO) and Kyoto Encyclopedia of Genes and Genomes (KEGG) pathway.

### Identification of the Association Between Prognostic and Ferroptosis-Related DEGs

To explore the relationship between ferroptosis and our prognostic genes, we first identified 60 ferroptosis-associated genes from the GeneCards (https://www.genecards.org/) (24), which contains up-to-date list of human ferroptosis-related genes. After merging samples, 142 glioma cases from the GEO database (GSE147352 and GSE59612) were qualified for subsequent analysis. Pearson’s test was performed to examine the correlation between ferroptosis-related DEGs and our prognostic gene expression.

### Immunological Analysis

Interactions between tumor and immune system were performed with the TISIDB database (25) and Xiantao tool (https://www.xiantao.love/products). We used the Xiantao tool to identify the demographic characteristics of MXRA8 expression and cancer immunology research in GBM, and then TISIDB was employed to cross-validate the roles of MXRA8 in immune-associated signaling pathways, such as tumor-infiltrating immune cells, and immunomodulators.

GEPIA2 is an interactive web server to analyze the RNA sequencing expression data of different cancer types and normal samples from the TCGA database and the GTEx projects, using a standard processing pipeline (26). Here, we computed the correlation between MXRA8 and immune monitoring sites in GBM and the corresponding normal tissues.

### Cell Cultures, Reagents, siRNA Transfections, and Macrophage Polarization

T98G and U251 human glioma cell lines were obtained from the Cancer Research Institute, Central South University, China, as described in our previous study (27); glioma cells were incubated in DMEM (C11995500, HyClone, Logan, UT, USA) supplemented with 10% fetal bovine serum (04-001-1A, BI, Israel) and 1% penicillin and streptomycin (10378016, Gibco, Waltham, MA, USA) at 37°C with 5% CO_2_. Temozolomide (TMZ) was purchased from Selleckchem (S1237, Berlin, Germany). Ras-selective lethal small molecule 3 (RSL3) and ferrostatin-1 (Fer-1) were purchased from APExBIO (B6095 for RSL3, A4371 for Fer-1, Houston, TX, USA). RSL3 and FER-1 were dissolved in DMSO under sterile conditions to storage concentration of 10 mM. Small interfering RNAs targeting MXRA8 and negative control siRNAs were synthesized by RiboBio (Guangzhou, China). As previously described (28), siRNAs were transfected into T98G and U251 cells for 48 h using Lipofectamine 3000 (L300015, Thermo Fisher Scientific, USA) according to the manufacturer’s protocol. To polarize M0 macrophages to M1, THP-1 cells (2.5 × 10^5^) were plated into 6-well plate and incubated with 320 nM phorbol 12-myristate 13-acetate (PMA; HY-18739, MedChemExpress, Monmouth Junction, NJ, USA) at 37°C for 6 h. To polarize M2 macrophages, cells were then treated with M2-polarizing reagents IL-4 (20 ng/ml; HY-P70445, MedChemExpress, USA) plus IL-13 (20 ng/ml; 200-13-10UG, Peprotech, USA) and incubated at 37°C for 72 h.

### CCK-8 Assays and IC50

T98G and U251 cells were seeded in 96-well plates (2 × 10^3^ cells/well) after transfection with siRNAs for 24 h. Various concentrations of TMZ (0, 0.39, 1.58, 6.25, 25, 100, and 400 μM) were added into the medium for 48 h. On the day of analysis, CCK-8 assays were conducted to examine cell viability following the manufacturer’s protocols. In Brief, 10 μl CCK-8 test solution (B34304, Bimake, USA) was added into each well and incubated for 1 h. Optical density (OD) of the samples was detected at 450 nm using a VICTOR X2 microplate reader (PerkinElmer, USA). Variable slope was conducted to calculate IC50 values using the Graphpad Prism8.0 software [nonlinear regression; dose-response inhibition; log (inhibitor) vs. response-variable slope (4 parameters)].

### Colony Formation Assay

The protocols used for the colony formation assay have been described previously (28). In brief, cells were transfected with siRNAs for 24 h and seeded in 6-well plates at a density of 1,000 cells per well. After incubating for 48 h, the cells were treated with different doses of TMZ. After incubation for approximately 14 days, colonies were stained with 0.3% w/v crystal violet/methanol for 20 min at room temperature.

### Iron Assay

Ferrous iron concentration was analyzed in T98G and U251 cells using an iron colorimetric assay kit (ab83366, Abcam, Cambridge, MA, UUSA) according to the manufacturer’s protocol. Briefly, cells were digested by trypsin without EDTA and were lysed in acid assay buffer at a density of 1 × 10^6^ cells per 1.5 ml tube. The samples were centrifuged at 14,000×*g* for 15 min to remove insoluble material. Ferene S was added to react with Fe^2+^, and a stable-colored complex was generated following the detection of absorbance at 593 nm by VICTOR X2 microplate reader (PerkinElmer, Waltham, MA, USA). The relative Fe^2+^ level was calculated as the ratio of concentration of all groups to concentration of siNC.

### Malondialdehyde Determination

Malondialdehyde (MDA) lipid peroxidation assay (MAK085, Sigma, St. Louis, MO, USA) was used according to manufacturers’ instructions. In brief, cells were collected by trypsin and lysed at a density of 1 × 10^6^ cells per 1.5 ml tube. Furthermore, lysis solution was reacted with thiobarbituric acid. Colorimetric assays were used to measure the absorbance at 532 nm by VICTOR X2 microplate reader (PerkinElmer, USA). The relative MDA level was calculated as the ratio of concentration of all groups to concentration of siNC.

### Western Blot Analysis

Cells were collected and lysed with RIPA lysis buffer with protease inhibitor cocktails (B14012, Bimake, Houston, TX, USA) at a ratio of 100:1 (v/v). The 50-µg protein extracts were equally loaded on 10% or 12% SDS-PAGE and then transferred to PVDF membranes (0.22 µm: ISEQ00010; 0.45 µm: IPVH00010). Following blocking with 5% skimmed milk for 1 h at room temperature, then membranes were incubated with primary antibodies diluted in 5% bovine serum albumin (D620272, Sangon Biotech, Shanghai, China) overnight at 4°C. Primary antibodies including MXRA8 antibody (1:1,000; ab185444, Abcam), NCOA4 antibody (1:1,000; ab86707, Abcam), FTH1 antibody (1:1,000; 4393, Cell Signaling Technology, Danvers, MA, USA), and GAPDH (1:10,000; 60004-1-Ig, Proteintech, Rosemont, IL, USA). For detection, the membranes were visualized by Immobilon Western chemiluminescent reagents (WBKLS0500, Millipore, Burlington, MA, USA).

### M2 Macrophage Infiltration Assays

The M2 macrophage infiltration assays were performed as previous described (21). In brief, 2.5 × 10^5^ M2 macrophage cells were seeded without serum for 12 h in the upper chamber of a Transwell plate (size 8 mm, Corning, Corning, NY, USA), while 2.5 × 10^5^ T98G and U251 glioma cells were incubated with10% FBS in bottom plate. After coincubation at 37°C for 24 h, the cells in the upper chamber were fixed in 4% formalin and stained with 0.3% crystal violet. The infiltrated M2 macrophage cells were counted in three randomly selected fields from each membrane.

### Immunohistochemistry

The glioma and normal brain tissues were obtained from the Department of Pathology, Xiangya Hospital. The tissues were paraffin embedded and the immunohistochemistry (IHC) was performed as previously described (29, 30). In brief, IHC was conducted using a Histomouse SP Kit (959551; Invitrogen, Waltham, MA, USA). Paraffin sections were immunostained through a streptavidin peroxidase procedure after microwave antigen retrieval. The signal was detected using a 3,3′-diaminobenzidine solution. The antibodies against MXRA8 (1:100, ab185444) and CSF1R (1:100, ab183316) were all purchased from Abcam. Images of the sections were independently examined and differentially quantified by two pathologists. IHC intensity score was scored as 0 (negative), 1 (weak brown), 2 (moderate brown), or 3 (strong brown). The extent of staining was scored as 0 (≤10%), 1 (11%–25%), 2 (26%–50%), 3 (51%–75%), or 4 (>75%). The final staining score was determined by multiplication of intensity scores and extent score and was classified as weakly positive (1–3), positive (4–6), and strongly positive (7–12). All paraffin-embedded specimens were collected following the ethical standards of the human experimental committee.

### Statistical Analysis

All experiments were conducted and repeated at least three times and were reported as the means ± SD. The differences between which were analyzed for significance using Student’s *t*-test for pairwise comparisons or ANOVA for multivariate analysis. Kaplan–Meier survival curves were estimated using the log-rank test to assess survival differences between groups. Data analysis was performed using GraphPad Prism 5 and SPSS 23.0. Differences were considered significant at ^*^p < 0.05, ^**^p < 0.01, and ^***^p < 0.001 for all tests.

## Results

### Identification of Prognostic-Related Gene MXRA8

**Figure 1:**
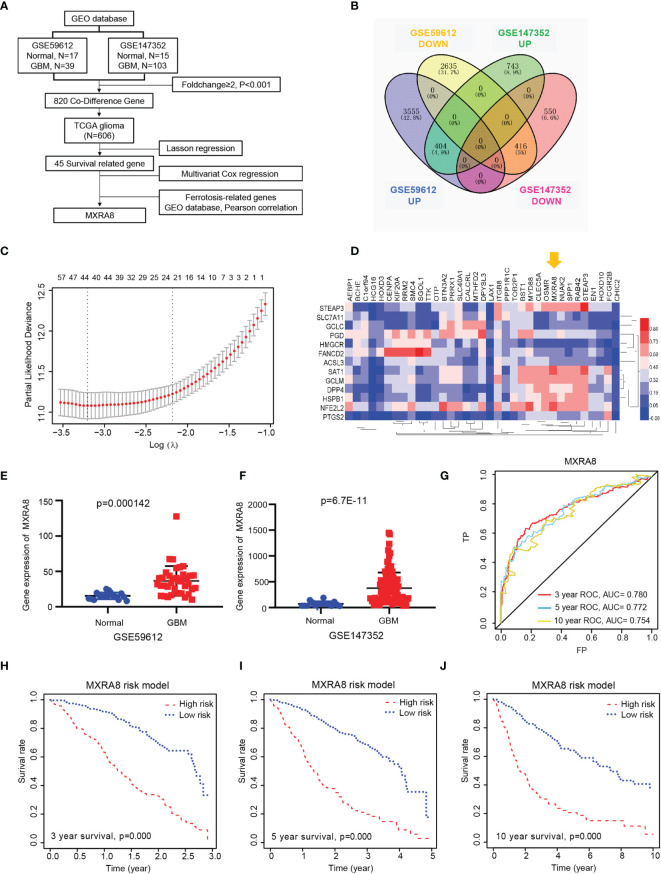
MXRA8 was identified as a novel prognostic-related gene in glioma. **(A)** Flowchart presenting the process of identifying the gene and prognostic nomogram of glioma in this study. **(B)** Venn diagram to identify differentially expressed genes between glioma and normal brain tissue from the GEO database. The 404 overlapping genes were all upregulated in tumor tissue. The 416 overlapping genes were all downregulated in tumor tissue. **(C)** Prognostic genes were identified using the least absolute shrinkage and selection operator Cox regression model (LASSO). The generated coefficient distribution plots for the logarithmic (lambda) sequence for the selection of the best parameter (lambda). **(D)** Heatmap of the correlations between 35 upregulated prognostic genes and the 14 ferroptosis-related DEGs. **(E, F)** The expression of MXRA8 was compared between normal and GBM tissue in the GSE59612 **(E)** and GSE147352 **(F)** cohorts. **(G)** AUC of time-dependent ROC curves verified the prognostic performance of the risk score based on MARA8 in the TCGA cohort. Three-year survival **(H)**, 5-year survival **(I)**, and 10-year survival **(J)** curves for the OS of patients in the high-risk and low-risk groups in the TCGA cohort.

The present study’s flow diagram is shown in **Figure 1A**. Two datasets from the GEO database (GSE147352 and GSE59612) were selected which have more than 50 samples. A total of 2,113 and 7,010 DEGs were identified between tumor and normal tissues, and 820 codifference genes were qualified for subsequent analysis (**Figure 1B**). Combined with the prognostic information, LASSON regression was then implemented to screen prognostic genes from the DEGs in the TCGA datasets. Finally, we found that 45 DEGs were significantly correlated with the OS of glioma patients (**Figure 1C**; **Supplemental Table S1**). Ferroptosis is an iron-dependent cell death with distinct properties and recognizing functions involved in cancers (31). The correlations between the 45 prognostic genes and 14 ferroptosis-related DEGs in the GEO dataset were analyzed in **Figure 1D** and **Supplementary Figure S1**. Interestingly, a receptor for multiple arthritogenic alphaviruses, MXRA8, which showed the highest correlation with ferroptosis may be involved in glioma progression and prognosis. GSE59612 and GSE147352 datasets respectively showed that expression of MXRA8 was significantly higher in gliomas than those in normal tissues (**Figures 1E, F**). Furthermore, to determine the prognostic effect of MXRA8, time-dependent ROC was applied to determine the prognostic values of the MXRA8-based risk scores. The AUCs for 3-, 5-, and 10-year overall survival predictions for the risk scores were 0.780, 0.772, and 0.754, respectively (**Figure 1G**). Patients from the TCGA dataset were stratified into two (high-risk and low-risk) groups. The survival curves revealed significantly favorable overall survival in all groups with lower-risk scores (*p* = 0.000) both in 3, 5, and 10 years (**Figures 1H–J**).

### Validation of the Expression Level and Clinical Significance of MXRA8

**Figure 2:**
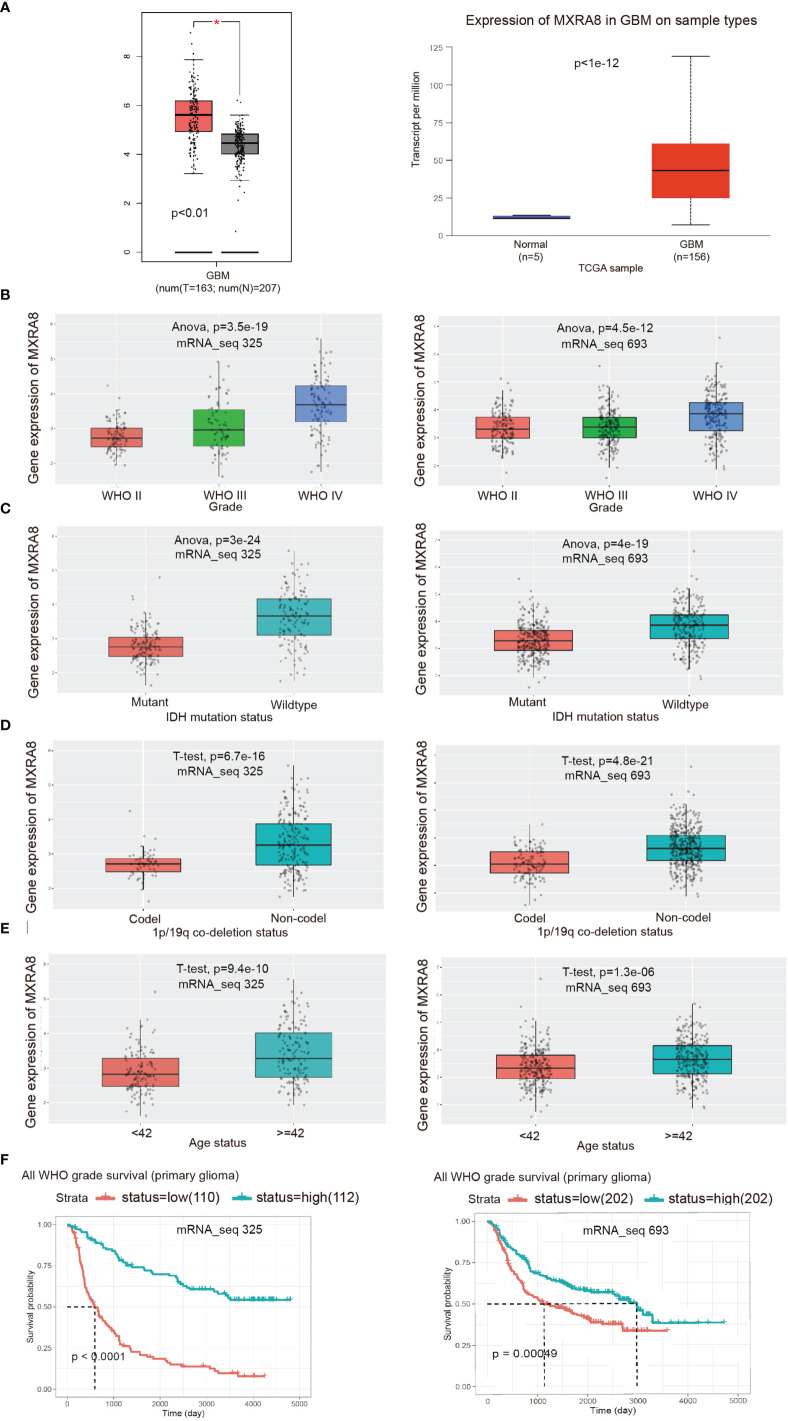
Validation of the expression level and clinical significance of MXRA8. **(A)** Expression comparisons of MXRA8 between glioma and normal control from GEPIA2.0 and UALCAN platforms. The “asterisk” represents *p* < 0.01. **(B–F)** The correlation of MXRA8 expression level with WHO grade **(B)**, IDH status **(C)**, 1p/19q codeletion status **(D)**, ages **(E)**, and survivals **(F)** from the CGGA database.

To further validate the role of MXRA8 in the malignant progression of glioma, we analyzed its expression levels in the TCGA datasets from the GEPIA2.0 and UALCAN platform, respectively. As shown in **Figure 2A**, MXRA8 expression levels were higher in glioma than those in normal specimens. We also detected the expression of MXRA8 in different grades of glioma from two CGGA datasets (RNAseq_325 and RNAseq_693) and observed that MXRA8 was obviously elevated in high-grade glioma (**Figure 2B**). IDH mutation and 1p/19q codeletion are distinctive indicators in glioma, with a tendency of favorable prognosis (32). We then explored the association between MXRA8 expression and the status of IDH and 1p/19q. MXRA8 expression significantly increased in patients with wild-type IDH and noncodel 1p/19q (**Figures 2C, D**). Additionally, patients over 42 years old showed higher MXRA8 expression (**Figure 2E**). The lower expression of MXRA8 was correlated with better prognosis of gliomas (**Figure 2F**). Furthermore, the demographic characteristics of MXRA8 from the Xiantao tool were in satisfactory concordance with the above results (**Table 1**).

**Table 1:** The demographic characteristics of MXRA8 expression in glioma.

Characteristics	Total (*N*)	Odds ratio (OR)	*p*-value
WHO grade (G4 vs. G2 and G3)	635	10.482 (6.656–17.150)	<0.001
1p/19q codeletion (noncodel vs. codel)	689	4.732 (3.207–7.117)	<0.001
Primary therapy outcome (PD vs. CR and PR and SD)	462	1.682 (1.091–2.592)	0.018
IDH status (WT vs. Mut)	686	10.629 (7.273–15.836)	<0.001
Histological type (glioblastoma vs. astrocytoma and oligoastrocytoma and oligodendroglioma)	696	9.529 (6.084–15.516)	<0.001
Gender (male vs. female)	696	1.098 (0.813–1.484)	0.540
Age (>60 vs. ≤60)	696	3.237 (2.182–4.882)	<0.001
Race (White vs. Asian and Black or African American)	683	0.920 (0.502–1.676)	0.784

### The Coexpression Network of MXRA8 in Glioma

**Figure 3:**
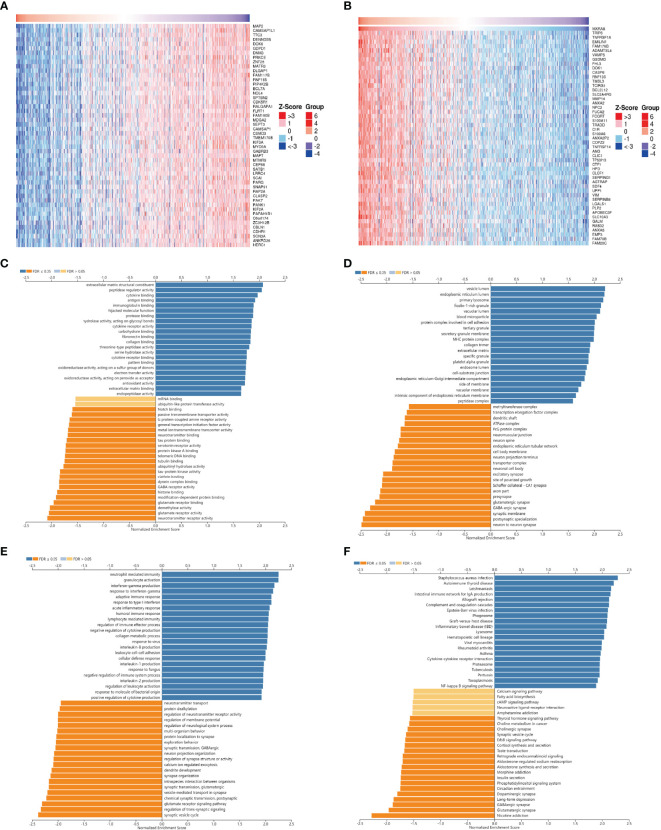
The co-expression network of MXRA8 in glioma. **(A, B)** Heatmaps of top 50 genes positively and negatively related to MXRA8. Red represents positively linked genes and blue represents negatively linked genes. **(C–F)** GO terms for molecular functions, cellular component, biological process, and KEGG pathways of MXRA8 by GSEA analyses.

In order to explore the biological functions of MXRA8 in glioma, we performed the coexpression pattern of MXRA8 screened from the TCGA-GBMLGG cohort by the LinkFinder module of LinkedOmics. **Figures 3A, B** shows the heatmaps of top 50 genes positively and negatively correlated with MXRA8, respectively. Additionally, we further performed GO and KEGG functional enrichment analyses to elaborate the potential biological roles of MXRA8 in GBM through the LinkInterpreter module. GO enrichment analysis mainly includes three parts: molecular function (MF), cellular component (CC), and biological process (BP). GO-MF enrichment analysis revealed that co-expression genes of MXRA8 mainly participated in extracellular matrix structural constituent, peptidase regulator activity, cytokine binding, antigen binding, immunoglobulin binding, etc. (**Figure 3C**). GO-CC analysis showed that these genes were significantly enriched in vesicle lumen, endoplasmic reticulum lumen, primary lysosome, ficolin-1-rich granule, vacuolar lumen, and so on (**Figure 3D**). GO-BP enrichment analysis suggested that these genes were significantly related to neutrophil-mediated immunity, granulocyte activation, interferon-gamma production, response to interferon-gamma, adaptive immune response, etc. (**Figure 3E**). KEGG pathway analysis indicated these genes were significantly related to inflammation and immune-associated signaling pathways, such as *Staphylococcus aureus* infection, autoimmune thyroid disease, leishmaniasis, intestinal immune network for IgA production, allograft rejection, etc. (**Figure 3F**). These findings suggested that MXRA8 might be involved in network of immunity-related functions in glioma.

### Knockdown of MXRA8 Inhibits Proliferation and Enhances the Sensitivity of TMZ in Glioma

**Figure 4:**
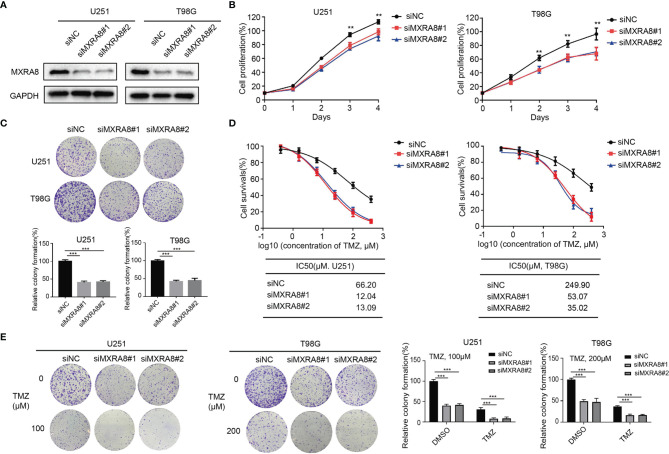
Knockdown of MXRA8 inhibits proliferation and increased the sensitivity of TMZ of glioma. **(A)** After U251 and T98G cells were transfected with siMXRA8 or siNC for 48 h, the protein expression of MXRA8 was analyzed by Western blot assay. **(B)** T98G and U251 cell proliferation was examined after treating with siMXRA8 or siNC for 5 days. **(C)** The colony formation was analyzed in T98G and U251 cells after treating with siMXRA8 or siNC. **(D)** Cell viability assay was performed in U251 and T98G cells to evaluate cytotoxicity of TMZ after transfection of siMXRA8. **(E)** The colony formation assay was conducted to examine after incubation with TMZ in U251 and T98G cells transfected with siMXRA8 or siNC. ^**^p < 0.01; ^***^p < 0.001.

To further examine the biological effect of MXRA8 in glioma, we knocked down MXRA8 with two small interfering RNAs in T98G and U251 cell lines (**Figure 4A**). Cell growth was significantly decreased in siMXRA8 compared with siNC (**Figure 4B**). Likewise, in colony formation assays, cell proliferations were significantly inhibited after transfection of siMXRA8 in T98G and U251 cells (**Figure 4C**). Overcoming the resistance of TMZ has been a vital problem in glioma treatment (33). To confirm whether MXRA8 could influence TMZ sensitivity in glioma, T98G and U251 cells were transfected with MXRA8 siRNA or siNC, followed by temozolomide incubation. Upon knockdown of MXRA8, T98G and U251 cells both showed increased sensitivity to TMZ, which manifested as reduced cell proliferation rates and about a 6-fold inhibition in IC50 (**Figure 4D**). Similarly, MXRA8 reduction markedly decreased the colony formation rates of T98G and U251 cells after treatment of 200 and 100 μM TMZ, respectively (**Figure 4E**). Collectively, these results suggest that downregulation of MXRA8 improved the sensitivity of glioma to TMZ.

### The Role of MXRA8 in Ferroptosis Regulation of Glioma

**Figure 5:**
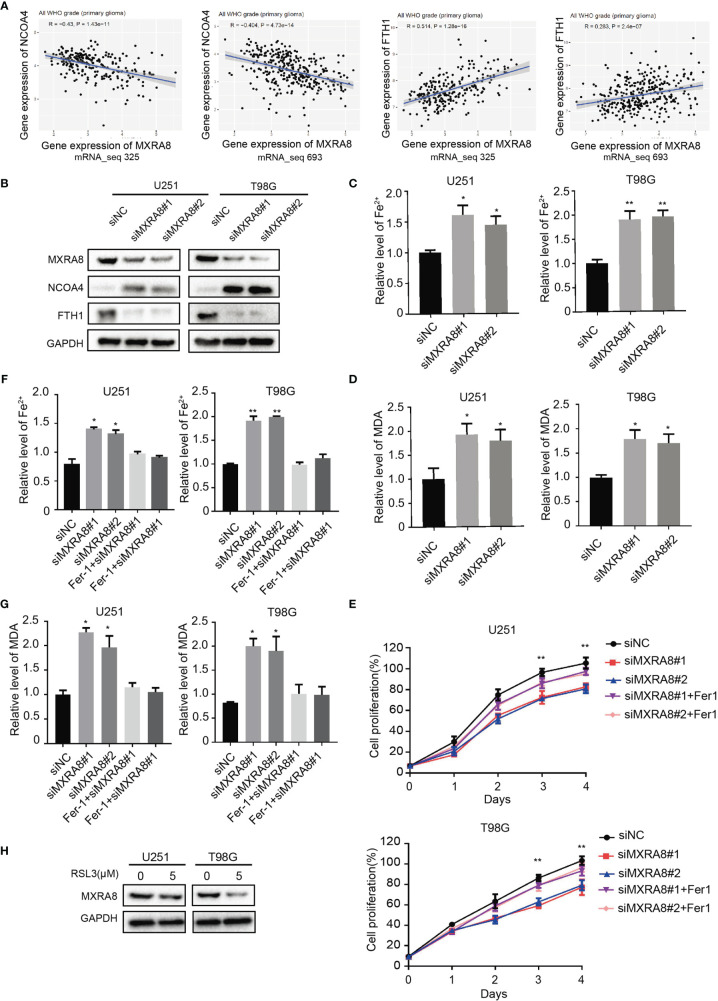
MXRA8 regulates ferroptosis in glioma. **(A)** The correlation between expression of MXRA8 and ferroptosis-related proteins including NCOA4 and FTH1. **(B)** NCOA4 and FTH1 were examined by Western blot in U251 and T98G cells transfected with siNC or siMXRA8. **(C, D)** Fe^2+^ and MDA levels were detected in U251 and T98G cells transfected with siNC or siMXRA8. **(E)** Cell viability was measured in siNC- or siMXRA8-transfected cells after pretreatment with RSL3 (5 μM) and subsequent Fer-1 (20 μM). **(F, G)** Fe^2+^ and MDA levels detected in siNC- or siMXRA8-transfected cells pretreated with RSL3 (5 μM) and Fer-1 (20 μM) for 24 h, successively. **(H)** After treatment of 5 μM of RSL3 in U251 and T98G cells, Western blot assay were performed to examine the protein expression of MXRA8. ^*^p < 0.05; ^**^p < 0.01.

The correlations between the 45 prognostic genes and 14 ferroptosis-related DEGs in the GEO dataset are shown in **Figure 1D** and **Supplementary Figure S1**. Pearson’s correlation coefficient indicated ferroptosis-related genes including DPP4, GCLM, HSPB1, NFE2L2, SAT1, STEAP3, and TFRC may be involved in MXRA8 function (Pearson’s *r >*0.5). To elucidate the molecular mechanism of MXRA8, the correlation between MXRA8 expression and ferroptosis markers, NCOA4 and FTH1 were performed using the two CGGA datasets (mRNA_seq 323 and 693). The expression of MXRA8 was positively associated with levels of NCOA4 and was negatively related to levels of FTH1. Also, the protein expression level of NCOA4 was increased in MXRA8 knockdown glioma cells, while FTH1 was reduced (**Figures 5A, B**). Ferroptosis is a process characterized by increased intracellular Fe^2+^ and lipid peroxidation, and the final product of lipid peroxidation is MDA. After MXRA8 knockdown, the intracellular Fe^2+^ and MDA levels were significantly increased by approximately 2-fold in T98G and U251 cells compared with controls (**Figures 5C, D**). To further understand the role of MXRA8 in cell proliferation and ferroptosis, Fer-1, a small molecule that serves as a ferroptosis inhibitor (34), was treated after MXRA8 knockdown. The results showed that Fer-1 could rescue the effects of cell growth inhibition induced by MXRA8 knockdown (**Figure 5E**). Moreover, Fer-1 reversed the increase of intracellular Fe^2+^ and MDA induced by downregulated MXRA8 (**Figures 5F, G**). Also, the protein expression of MXRA8 was reduced after treatment of ferroptosis agonist RSL3 (**Figure 5H**). Taken together, these data indicated that knockdown of MXRA8 could suppress cell survival and enable ferroptosis in glioma cells.

### The Role of MXRA8 in Immune Mircoenviroment of Glioma

**Figure 6:**
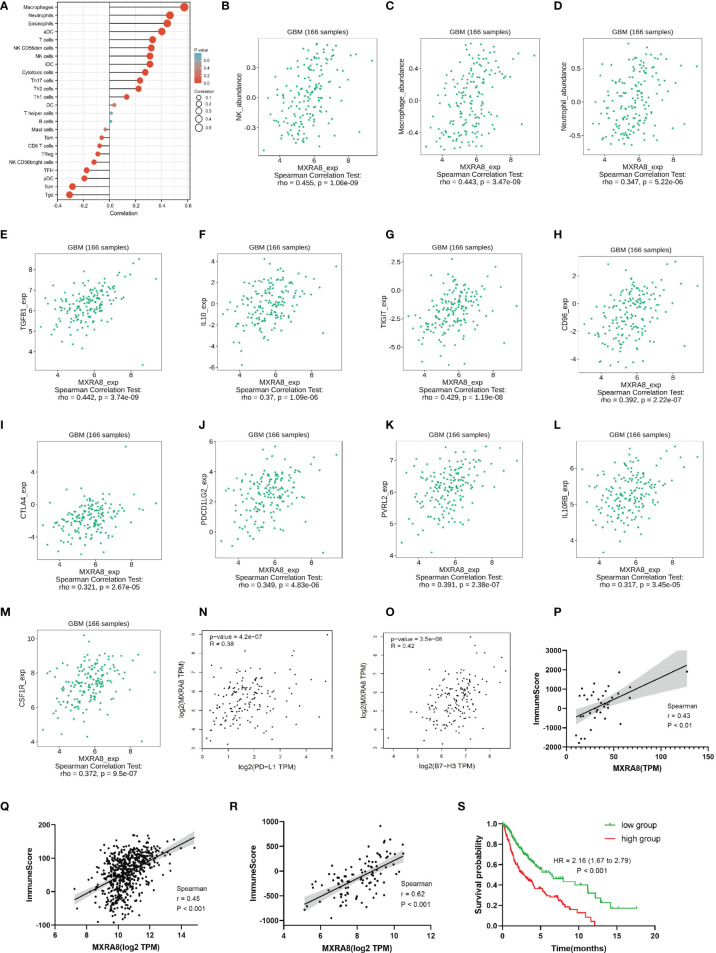
The role of MXRA8 in immune microenvironment of glioma. **(A)** Immune infiltration cells associated with MXRA8 using ssGESA algorithm from the TCGA database. **(B–D)** Validations of NK cells, macrophages, and neutrophils by the TISIDB database. **(E–O)** Associations of the MXRA8 expression with chemokines **(E, F)** and multiple immunoinhibitory molecules **(G–O)** from the TISIDB database. **(P–R)** The association between MXRA8 with immune score calculated by ESTIMATE algorithm, based on GSE59612, GSE147352, and TCGA glioma datasets. **(S)** The Kaplan–Meier curve of immune score on overall survival of TCGA glioma datasets.

Increasing evidence has revealed the association between ferroptosis and immune microenvironment (35). Therefore, we further explored the role of MXRA8 in the immune microenvironment of glioma. The correlations between MXRA8 and various immune infiltration cells were analyzed by Xiantao tool using ssGSEA algorithm. Natural killer cells (NK), macrophages, and neutrophils were found to be positively correlated with MXRA8 in glioma (**Figures 6A–D**). Using TISIDB database, we further analyzed the associations between MXRA8 with immunoinhibitors. As shown in **Figures 6E, F**, indicated immunoinhibitors were found to have significant associations with the expression of MXRA8, while transforming growth factor beta 1 (TGFB1) showed the highest correlation coefficient. Moreover, **Figures G–O** shows the positive associations between MXRA8 expression levels and multiple immune checkpoint molecules, including PD-L1 and B7-H3. In addition, **Figures 6P–R** exhibits a positive correlation between MXRA8 and immune infiltration score in GSE59612, GSE147352, and TCGA glioma datasets. We further investigated the prognostic value of the immune infiltration score in the TCGA glioma dataset, and the results showed that patients with higher immune infiltration score had a poorer OS (**Figure 6S**). These results together indicated that MXRA8 might influence the prognosis of glioma patients through regulating the immune response.

### The Effects of MXRA8 on M2 Macrophage Infiltration

**Figure 7:**
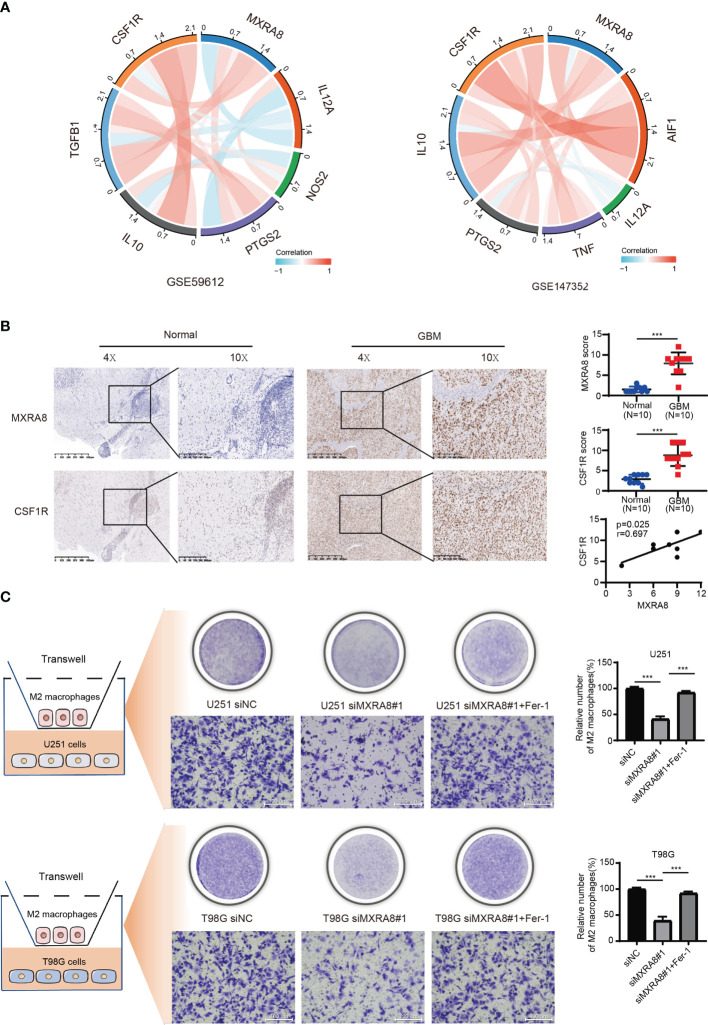
The effects of MXRA8 on M2 macrophage infiltration *in vitro* by mediating ferroptosis. **(A)** The correlation coefficient between MXRA8 and classical phenotype markers of macrophages in the GEO datasets. **(B)** The expression level of MXRA8 and a M2 macrophage phenotype marker (CSF1R) in GBM and normal brain specimens determined by IHC analysis. **(C)** Infiltration of M2 macrophages in transfected siNC or siMXRA8 cells (U251 and T98G) after pretreatment with RSL3 (5 μM) and subsequent Fer-1 (20 μM). ^***^p < 0.001.

The above results confirmed positive correlation between MXRA8 and macrophage infiltrations (**Figures 6A, B**) and cytokines (such as IL-10 and TGF-β1) that are involved in tumor-associated macrophage infiltration were highly consistent with expression of MXRA8. Through a correlation coefficient analysis performed in the GEO datasets (GSE59612 and GSE147352), we investigated the relationship between the expression of MXRA8 and the typical phenotype macrophage markers, including M (AIF1), M1 (IL12A, TNF, NOS2, PTGS2), and M2 (IL10, CCL163, TGFB1, CSF1R) (21) (**Figure 7A**; **Table 2**). The M2 macrophage markers CSF1R showed significantly positive correlation (cor_spearman >0.3, *p* < 0.05) with MXRA8 in both datasets. Using 10 paraffin-embedded specimens, IHC staining indicated the intensity of MXRA8 and CSF1R was stronger in GBM than normal. The staining intensity of macrophage M2 marker CSF1R was positively correlated with MXRA8 (Pearson’s *r* = 0.697, *p* = 0.025) (**Figure 7B**). To further investigate MXRA8 effect in ferroptosis and macrophage infiltration, THP-1 cells were differentiated into M2 macrophage, and U251 and T98G cells were treated with Fer-1 after knockdown of MXRA8. The results showed that downregulation of MXRA8 decreased the infiltration of M2 macrophage, while the reduced M2 macrophage infiltration generated by MXRA8 knockdown was rescued by Fer-1 treatment (**Figure 7C**).

**Table 2:** The correlation coefficient analysis between MXRA8 and phenotype macrophage markers in the GEO datasets.

GSE147352				GSE147352			
Gene name	Gene name	cor_spearman	*p*-value	Gene name	Gene name	cor_spearman	*p*-value
MXRA8	IL12A	−0.422	0.007	MXRA8	IL12A	−0.190	0.002
	NOS2	−0.011	0.948		TNF	−0.041	<0.001
	IL10	0.163	0.007		CSF1R	0.331	0.025
	PTGS2	0.343	0.322		PTGS2	0.350	<0.001
	CSF1R	0.412	0.016		IL10	0.471	<0.001
	TGFB1	0.491	0.038		AIF1	0.557	<0.001

## Discussion

In this work, we aimed to investigate critical and novel indicators for prognosis of glioma. Through GEO datasets and TCGA database, 45 DEGs were screened, as these genes were strongly associated with OS of glioma patients. Intriguingly, among the 45 DEGs, MXRA8 was screened as an indicator of poor prognosis with malignancy clinical characteristics of malignancy including IDH wild type and 1p/19q noncodeletion. Furthermore, we demonstrated that knockdown of MXRA8 inhibited cell proliferations *in vitro* and contributed to increasing sensitivity of glioma cells to TMZ. These data firstly provide a novel perspective of MXRA8 which could serve as a prognostic indicator in glioma.

Emerging evidence showed that ferroptosis could be triggered by several signal pathways and/or small molecular compounds in glioma. For instance, dihydroartemisinin was identified to trigger ferroptosis accompanied with ROS generation and lipid peroxidation (36). Furthermore, erastin could sensitize GBM cells to temozolomide by restraining SLC7A11 and cystathionine-γ-lyase function (37). This evidence suggests that targeting the pathway of ferroptosis could bring out the new strategies for glioma treatment. In our studies, we firstly found that downregulation of MXRA8 could induce ferroptosis by elevating the Fe^2+^ and MDA levels in glioma cells. Furthermore, we observed a significant correlation between MXRA8 expression and ferroptosis-related marker NCOA4 and FTH1 according to the CCGA datasets. NCOA4 is a selective cargo receptor for the selective autophagic turnover of ferritin in ferroptosis, while ferritin heavy polypeptide 1 (FTH1) is a subunit of ferritin. Since degradation of ferritin could elevate iron levels and generate cellular oxidative stress by the Fenton reaction (38), it has been proven that NCOA4 could bind up to FTH1 and thus deliver ferritin to the lysosome for degradation (39). In glioma, a study reported that NCOA4 caused degradation of ferritin and a subsequent increase in levels of Fe^2+^and ultimately ferroptosis (40). Likewise, our study also demonstrated that inhibition of MXRA8 increased the protein expression of NCOA4 and decreased FTH1 protein levels in glioma cells. These above results could help us further understand the potential role of MXRA8 in mediating ferroptosis.

Multiple studies have revealed that immunosuppressive microenvironment was a nature of glioma, which further inhibits antitumor immune responses (41). An amount of immune cells, chemokines, and checkpoints were recognized as biomarkers of immune evasion and tumor progression. Glioma-associated macrophages could secrete cytokines such as IL-10 and TGF-β, which decrease the activities of immune cells and promote tumorigenesis (42, 43). Glioma-associated neutrophil infiltration which promoted tumor growth and resistance to antiangiogenic therapies was considered an indicator of poor prognosis (44, 45). Moreover, glioma cells express increased levels of immunosuppressive factors such as PD-L1, TIGIT, CD96, and CTLA-4, which negatively regulate presentation of antigens and T-cell responses (46–48). Especially, expression of PD-L1 could respond to the presence of T-cell-generating cytokine interferon gamma and trigger reduction of T-cell killing capacity, which further caused adaptive immune resistance (49, 50). In the present study, the results showed MXRA8 might participate in neutrophil-related immunity, adaptive immune response, and cytokine binding. Furthermore, our studies revealed that MXRA8 expression was positively associated with levels of these immunoinhibitory factors involved in microenvironment of glioma. Additionally, immune infiltration score in the TCGA glioma datasets showed that patients with high immune infiltration had a tendency with upregulation of MXRA8, which couples with worse OS. These results altogether imply that the expression of MXRA8 might be parallel to immunosuppression and developed as a therapeutic target of immunotherapy in glioma.

The role of macrophages in tumor growth was complex in dependence on various factors and might be polarized to antitumor (M1) or protumor (M2) phenotypes (51). Targeting M2 macrophages in the glioma microenvironment could become an alternative therapeutic strategy. CSF1R-regulated signaling is crucial for the differentiation of M2 macrophage polarization in various cancers, and blockade of CSF1R could convert macrophage polarization and inhibit glioma progression (52, 53). In our study, we found that CSF1R was positively correlated with MXRA8 expression in GBM, which suggested that functional activity of MXRA8 might be related to regulation of M2 macrophages. Furthermore, several studies have reported that ferroptosis-related gene signature promotes tumor progression by influencing macrophage infiltration in various cancers (54, 55), but the biological roles of ferroptosis in glioma-associated macrophages were still unclear. Our present study firstly demonstrated that knockdown of MXRA8 could inhibit M2 macrophage migration to glioma cells through regulation of ferroptosis. These findings indicate that targeting MXRA8-related ferroptosis might become a promising strategy for immunotherapy.

From now on, the integration between ferroptosis and immunity environment remains unclear, especially in glioma. Our study uncovers the essential roles of MXRA8 in glioma progression and highlights the importance of MXRA8 in regulating ferroptosis and immune microenvironments. We identified that MXRA8 supersession could enable ferroptosis activation, and a hint of MXRA8 in regulation of immune microenvironment was provided. Therefore, our findings suggest that MXRA8 could be a promising prognostic biomarker in patients with glioma.

## Data Availability Statement

The datasets presented in this study can be found in online repositories. The names of the repository/repositories and accession number(s) can be found in the article/**Supplementary Material**.

## Ethics Statement

The studies involving human participants were reviewed and approved by the ethics committee of Xiangya Hospital, Central South University. Written informed consent for participation was not required for this study in accordance with the national legislation and the institutional requirements.

## Author Contributions

ZX and YY contributed to the conception and design of the study. YY and XC contributed to the writing, review, and/or revision of the manuscript. XC, YF, and LS provided administrative, technical, or material support. All authors contributed to the article and approved the submitted version.

## Funding

This study is supported by grants from the China Postdoctoral Science Foundation (2021T140754, 2020M672521), the Science and Technology Innovation Program of Hunan Province (2021RC3029), the Natural Science Foundation of Hunan Province (2020JJ5934), and the Postdoctoral Science Foundation of Central South University (248485).

## Conflict of Interest

The authors declare that the research was conducted in the absence of any commercial or financial relationships that could be construed as a potential conflict of interest.

## Publisher’s Note

All claims expressed in this article are solely those of the authors and do not necessarily represent those of their affiliated organizations, or those of the publisher, the editors and the reviewers. Any product that may be evaluated in this article, or claim that may be made by its manufacturer, is not guaranteed or endorsed by the publisher.

## Supplementary Material

Supplementary Table 1 45 DEGs were significantly correlated with the OS of glioma patients.

Supplementary Figure 1 Heatmap of the correlations between 10 downregulated prognostic genes and the 14 ferroptosis related DEGs.
