# Supplementary figures and images for: Matrix Remodeling-Associated Protein 8 as a Novel Indicator Contributing to Glioma Immune Response by Regulating Ferroptosis
Source: Front Immunol. 2022 Feb 24;13:834595. doi: 10.3389/fimmu.2022.834595 (PMC8911537; doi:10.3389/fimmu.2022.834595)

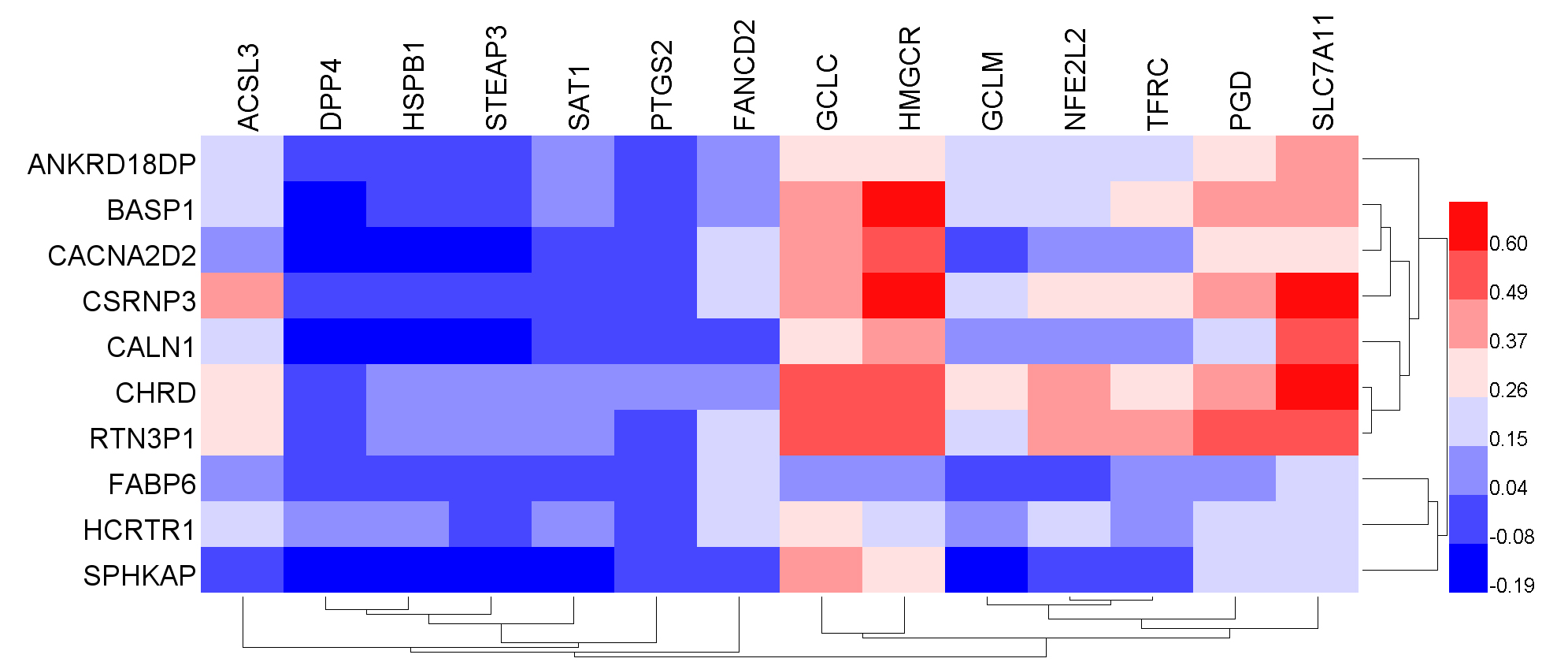

Supplement: Supplementary Figure 1 — Heatmap of the correlations between 10 downregulated prognostic genes and the 14 ferroptosis related DEGs. [file Image_1.jpeg]
